# Deep learning for medical image analysis: a brief introduction

**DOI:** 10.1093/noajnl/vdaa092

**Published:** 2021-01-23

**Authors:** Benedikt Wiestler, Bjoern Menze

**Affiliations:** 1 Department of Neuroradiology, TU Munich University Hospital, Munich, Germany; 2 Department of Informatics, TU Munich, Munich, Germany

**Keywords:** convolutional neural networks, deep learning, glioma, image analysis

## Abstract

Advances in deep learning have led to the development of neural network algorithms which today rival human performance in vision tasks, such as image classification or segmentation. Translation of these techniques into clinical science has also significantly advanced image analysis in neuro-oncology. This has created a need in the neuro-oncology community for understanding the mechanisms behind neural networks and deep learning, as close interaction of computer scientists and neuro-oncology researchers as well as realistic expectations about the possibilities (and limitations) of the current state-of-the-art is pivotal for successful translation of deep learning techniques into practice. In this review, we will briefly introduce the building blocks of neural networks with a particular focus on convolutional neural networks. We will explain why these networks excel at identifying relevant features and how they learn to associate these imaging features with (clinical) features of interest, such as genotype, or how they automatically segment structures of interest in the image volume. We will also discuss challenges for the more widespread use of these algorithms.

Advances in deep learning techniques (and the increasing availability of computing resources) have led to the development of algorithms which rival (or surpass) human performance in a wide range of applications, many of which were previously considered to be impossible to master for algorithms. This ranges from playing the game of Go^1^ to medical applications, such as classifying skin lesions^2^ or predicting future acute kidney injury in patients.^3^ Arguably, most progress has been achieved in vision tasks, where algorithms, and in particular convolutional neural networks (CNNs), began reaching human performance in classification tasks in 2012.^4^

Given the rich information on disease biology contained in medical images, these developments have attracted a lot of attention in the medical community. Subsequently, deep learning techniques have successfully been applied to all aspects of medical imaging, from image reconstruction^5^ to postprocessing^6^ and image analysis.^7^

For successful application of these powerful algorithms to research questions, close interaction of computer scientists and neuro-oncology researchers is pivotal. This also requires researchers to have an understanding of how deep learning (and in particular neural networks) function, how (and what) these algorithms learn, but also what limitations to their use exist.

## What Is a Neural Network, and How Do They Learn?

Neural networks represent a class of algorithms that are designed to recognize patterns, and which can help in clustering or classifying input data. Their design follows a structure which is loosely inspired by building blocks of information processing in the human brain, hence their name.

More formally, neural networks represent functions that map numerical input *x*, for example, a given image patch, to a corresponding predictor *y*, for example, an anatomical label for the central pixel of the patch. To do this, the neural network algorithm learns a function *f* that represents the observed correlation between *x* and *y* and the approximate mapping of *f*(*x*) = *y*. In this, they are similar to many other nonlinear regression and classification algorithms from the fields of machine learning and statistical learning.

What makes Neural Networks radically different from these “classic” approaches is their architecture. This architecture in general consists of many small nodes (or “neurons”), which are organized into several stacked layers. In 1958, Rosenblatt described an early, shallow version of this architecture, termed “perceptron”.^8^ If the number of the stacked layers (or “depth”) of the overall network becomes larger than a perceptron, that is, than what is required in theory for solving an arbitrary nonlinear classification or regression task, they are oftentimes referred to as Deep Learning architectures.

**Figure 1.**
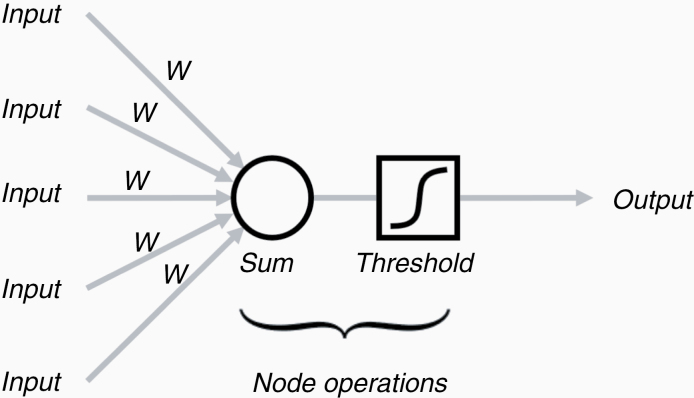
Calculations at a node. The computational operations at a node include a weighting of the input signals by factor *w*, summing over them, and determining whether this sum surpasses a critical threshold and turning the output signal from “off” to “on.” Often a logistic function is used instead of a hard threshold, and the overall operation is identical to a logistic regression. The weights and the parameters of the nonlinear “threshold” function are properties of the node that are optimized during the training phase.

**Figure 2.**
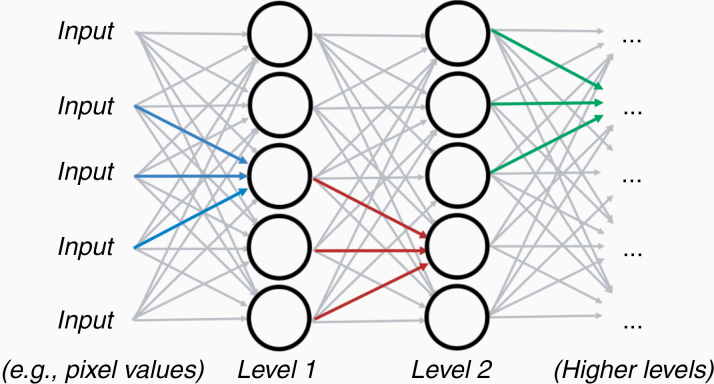
Neural Network architecture. A node layer or level represents a parallel set of individual nodes (here: circles) that turn on or off depending on the input they obtain from the previous layer. All nodes of a layer are computing in parallel, and then pass on their output signals to a subsequent layer. A multilayer perceptron would have connections between all nodes of 2 layers, while a Convolutional Neural Network (CNN) would only link nodes that are nearby (blue, red, and green), dropping most of the far reaching connections. However, this constrained set of weights, or “filter,” is reused in all nodes of a layer.

The building blocks of these networks are the nodes (Figure 1). A node multiplies the input from a lower layer with a set of coefficients, or weights *w*, that assign significance to those inputs that are relevant with respect to a prediction of *y*. The weighted input is summed and passed through a nonlinear function, which modifies the signal of the node and forwards it to the other nodes at the next level in case it supasses a critical threshold (Figure 2). This concept of a node is inspired by the architecture of neurons in the brain that gather and weigh input signals, and either switch “on” or remain “off” depending on how strong the input signals are. Formally, the node is similar to a logistic regression and, hence, an artificial neural network is similar to a collection of a high number of logistic regressions that are executed in parallel and stacked on top of each other.

Training a Neural Network is the process of finding those parameters of the nodes—which are primarily the weights *w*—that optimize the mapping of *f*(*x*) to *y*, that is, that minimize the error in the predicted *y*. At the node level, and less formally, the following is happening during training in 3 steps:

(1) Input *x* (eg, an image) is presented to the first node. The weights *w* of the node determine the contribution input *x* has to the output *p* of this node (see also Figure 1):

$$\begin{array}{l} x*w=p \end{array}$$ (1)

where *p* is an intermediate score calculated for the given input. In reality, many of these nodes are stacked and the output *p* of 1 node contributes to the input *x* of nodes in the following level.

(2) At the end of the network, a predicted output *y_pred* is generated from the scores *p*. The overall accuracy of the network is then evaluated by comparing the prediction with the ground truth *y*:

$$\begin{array}{l} y\_pred\;\text{-}\;y=error \end{array}$$ (2)

(3) This difference determines the global error the network *f*(*x*) makes in predicting *y*. In a next step, the error is propagated back from the final output toward the input layer, adjusting weights *w* in the layers in between in accordance to their contribution to the error:

$$\begin{array}{l} error\;*\;\mathrm{w\prime}s\;contribution\;to\;error=adjustment\;of\;w \end{array}$$ (3)

These 3 steps represent the full training and update strategy: (1) evaluate and score the input with a given set of parameters, (2) calculate the error or “loss,” and (3) use the information about that loss to update the model parameters.

So how does the network “know” how to adjust these weights? Updating the model parameters, which are in the number of millions even for small networks, poses a hard optimization problem that needs to be solved for each learning task anew. Randomly exploring parameters combinations therefore is not an option. Fortunately, a mathematical property of Neural Networks alleviates this procedure. Neural Networks have a derivative function that is straightforward to calculate, and that is indicating how the error changes in dependence to presumed changes in the model parameters *w*. Having this “derivative” or “gradient,” that determines the weight’s contribution to errors (Eq. (3)), makes a particularly powerful set of optimization algorithms applicable to the network adaptation task and simplifies the optimization of the model’s parameters dramatically. An optimizer with “gradient descent” does not have to explore the space of all possible combinations of values of the millions of network parameters *w*, but it explores the space of acceptable solutions of *w* in a very structured and well defined fashion. With every iteration of the 3 steps from above (Eqs. (1)–(3)), that is, with every update of weights according to their impact on the overall error, the optimization proceeds along a path in the parameter space of *w* that aims at a minimum until updates cannot reduce the error any more. This “gradient descent” allows even highly complex Neural Networks with millions of tunable parameters to learn efficiently.

## How Do Neural Networks “See” Images?

A regular Neural Network uses the same input as any other Machine Learning algorithm—an ordered set of features, for example, a vector containing a set of relevant clinical variables. This set of variables remains the same during training and prediction, the type of variable in a specific entry of the vector also has to remain the same. This makes a standard Neural Network difficult to use for image data. Oftentimes, the patterns of interest are not bound to a particular region of the image or specific entries of the image array. Moreover, they easily have millions of pixels and, hence, input values to the Neural Network, increasing the amount of parameters and the memory required during the training iterations significantly (and beyond usable limits).

**Figure 3.**
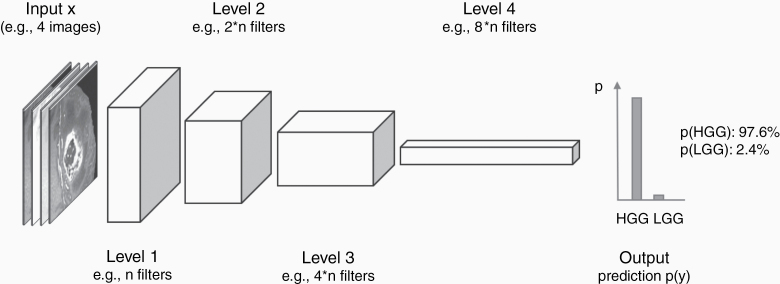
Image classification with CNNs. Classification CNNs perform a down-convolution from image size to single labels with increasing numbers of filter sets. The number of filters used is a hyperparameter of the network that can be defined and optimized. The final output is a global class label or its probability indicating, for example, whether the presented set of MR images show a low- or a high-grade glioma. CNNs, Convolutional Neural Networks.

To this end, many Neural Networks for image processing have 2 characteristic modifications: the entries of a node (Figure 3) are constrained to a few input signals from pixels or nodes in the direct spatial vicinity of the node itself, decreasing the number of parameters that need to be processed and estimated dramatically. As an additional constraint, to enforce spatial invariance of the pattern classifier, the entry parameters *w* are shared throughout all nodes at the same level, that is, they are applied to inputs of every single location alike. Mathematically, this is equivalent to “convolving” the image with *w* after grouping the weights of *w* in a local “filter.” Since using only one such filter at a level may result in too much information being filtered out, there are often several dozens or hundreds of filters used and learned in parallel. As all these image-specific Neural Networks make use of filter sets that are applied by means of image convolution, they are all referred to as CNNs.

CNNs are successful in a wide range of image processing tasks. Still, it remains a challenge to understand the internal information processing of a network. Some studies attribute their high performance to the hierarchical organization of the network: filters in the lower level seem to resemble standard local image filters, for example, enhancing edges under specific orientations, while filters in higher levels are increasingly specific about individual components of the structures to be detected, until only one global label is predicted as the final output at the top of the network. This property has a highly desirable consequence that contributes to the popularity and performance of Deep Learning methods: The image filters that for “classical” machine learning algorithms need to be chosen during the design process can now directly be learned from the data during training. To this end a consultation with an image processing specialist is not required any more for choosing optimal filters. Moreover, as the lower levels of the network are populated with rather generic feature extractors, it becomes possible to start the training and the optimization of the network’s weights *w* not from arbitrary values, but from values that have been found to work well in a related task. This allows, for example, that an algorithm for detecting anatomical structures of interest in an X-ray image can be almost identical to one that has been developed for detecting objects, such as cars or trees or animals. Only a few modifications of the final task-specific layers in the network may be necessary in “fine tuning” during training, if the lower layers of the new network have been prepopulated or initialized with parameters *w* from the previously available network. This successful reuse and transfer of networks trained by other researchers on other tasks, where data or computing resources might have been easily available, is another major reason for the popularity of neural networks in image processing tasks.

To further strengthen the algorithm’s invariance to the spatial localization of the signal to be detected, and to further decrease the amount of parameters of the network, many CNNs downsample the number of nodes at each level after, for example, averaging output signals of a layer before they are used as input to the next one (Figure 3). This leads to networks that have as many nodes as pixels at the entry level, then consistently downsample the output of the convolutional filters at each level, to only have one last global predictor at the end. These “down-convolutional” networks have exceptional performances for image classification tasks, that is, when inference is about estimating this one predictor at the end.

**Figure 4.**
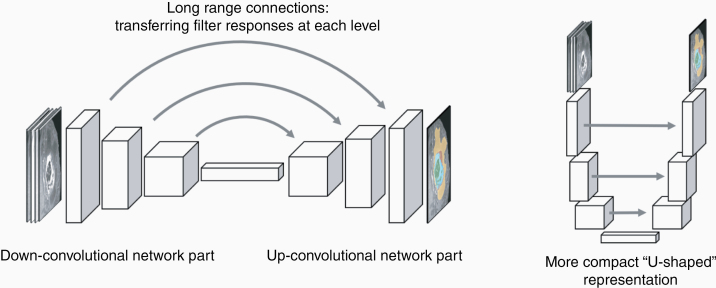
Image segmentation and “U-Net” architectures. For segmentations, the downsampling taking place in a global classification has to be inverted to have predictions about class memberships at the pixel level. To this end, the network of Figure 3 is “mirrored” and an “up-convolutional” part is added. To enhance performance, the filter maps of the down-concolutional part are also directly passed on to the up-convolutional arm of the network (gray arrows). When rearranged in a more compact representation that emphasizes these “long-range” transfers between the levels, a U-shape is obtained that is characteristic for these “U-Net” CNN segmentation architectures. CNNs, Convolutional Neural Networks.

Still, having a global label that indicates, for example, that a tumor is present in the image, does not inform about where this tumor may be in a given image patch, and it cannot be used for delineating or segmenting the tumor. To this end, most CNN-based segmentation algorithms do not only rely on architecture that downsample images and filters, but that have another upsampling or “up-convolutional” component to return a map of predictors that has the same size as the input and, hence, one labels for each pixel (Figure 4). In such a network, the downsampling essentially serves as a global detector of the structure interest, while the upsampling is used to identify the location where the detected structure, for example, the brain tumor, is present in the image. As the downsampling filters the image information heavily, and some of the information that is relevant for solving the subsequent localization task is not passed on and lost, intermediate filter results from the down-convolutional side of the network are passed over to the up-convolutional side (Figure 4, left). As the resulting network architecture can be well described by a “U” shape, segmentor networks are often referred to as “U-Net” ^9^ (Figure 4, left).

## How Can Neural Networks Use (My) Data More Efficiently?

A common argument against building Neural Networks is that they are perceived to require large training datasets. While it is true that Deep Learning techniques can effectively be trained on vast amounts of data (Chilamkurthy et al. trained a head computed tomography (CT) classifier on more than 300 000 CT scans^10^), Neural Networks typically outperform “classic” machine learning models even when trained on small dataset (although more data will yield better results). In their seminal paper on the U-Net, Ronneberger et al. significantly outperformed the prior state-of-the-art algorithms for segmenting microscopy images with training datasets of only 35 and 20 partially segmented images, respectively.^9^ Several strategies exist to help neural Networks utilize data more efficiently (image augmentation and transfer learning in particular).

**Figure 5.**
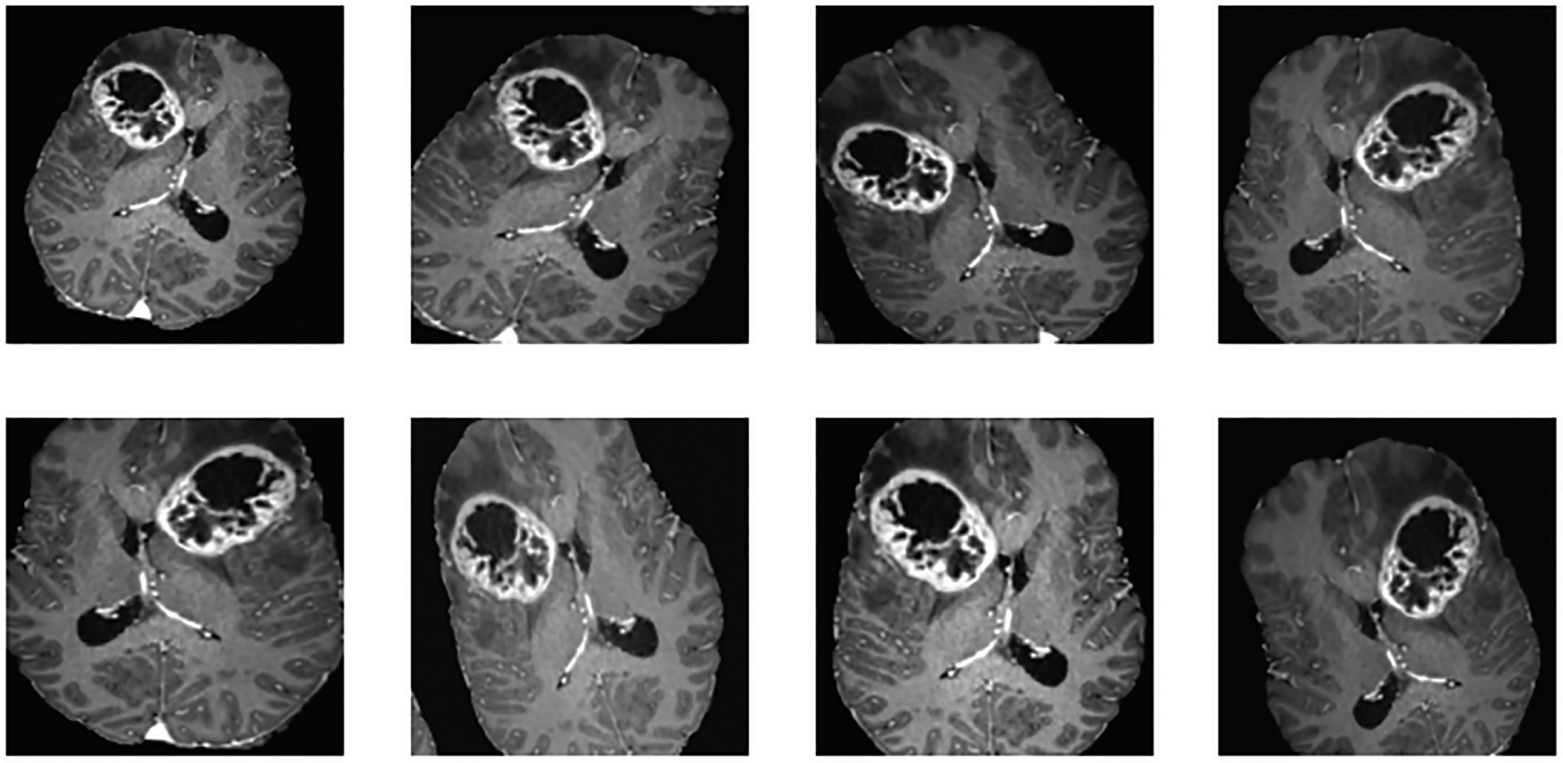
Example of image augmentation. In image augmentation, random combinations of simple operation like flipping, scaling, or shifting images introduce variations in the input data. These small variations lead to more robust models, which have a higher invariance against rotation and scaling in the input data.

**Figure 6.**
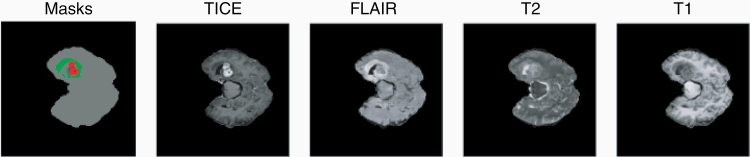
Example of synthetic images used for data augmentation. The MR images are created synthetically from a given input segmentation mask (left), allowing for efficient enrichment of image datasets. Image taken from Red-GAN.^13^ T1CE, contrast-enhanced T1.

With the 2-fold aim of enriching the training dataset and at the same time building more robust Neural Networks, image augmentations is today commonly used during Neural Network training. The idea behind image augmentation is to apply random changes (or combinations thereof) to the input images, such as flipping or scaling the images, adding noise or standardizing intensities. An example of these operations is shown in Figure 5. These small variations in the input images have been shown to both increase performance as well as making models invariant to image orientation or scale.^11^ This latter aspect is particularly attractive in medical imaging, where models must be invariant to the random shapes seen in pathologies. With the introduction of generative adversarial networks (GAN),^12^ which allow the synthesis of images, more sophisticated image augmentation strategies are currently being developed. Qasim et al. for example have developed a GAN which is able to generate realistically looking glioma images from segmentation masks (Figure 6).^13^

Another important technique to improve network performance is transfer learning. In transfer learning, a network which has been pretrained on a large dataset (typically from a different domain such as natural images) is selectively fine-tuned for the task of interest using the (typically small) training dataset. The image filters which have been prelearned on the initial task are thereby efficiently retrained: those in the lower levels—that resemble standard local image filters—are almost identical for a wide range of tasks; and only few of them—that are specific to the given detection task—need to be readjusted in the higher levels, where down-convolutions has already reduced their numbers significantly. Compared with being trained from scratch, pretraining networks for medical image analysis tasks have been shown to improve performance in a variety of applications such as detecting diabetic retinopathy in retinal fundus photographs or predicting Alzheimer’s disease in fluorodeoxyglucose positron emission tomography, even when the networks were pretrained on natural images.^14,15^ Raghu et al. provide a more in-depth analysis of the effects of transfer learning.^16^

One main fear with training complex models such as Neural Networks with small amounts of data is that these models will essentially “memorize” the training data, that is, become overfitted. In classical machine learning, overfitted models tend to perform poorly on unseen test data. Neural Networks however often show good performance on test data, even when they are trained to exactly fit. In a recent paper, Belkin et al. propose that the “bias-variance-tradeoff” known in classical machine learning takes a different form in Neural Networks.^17^ They argue that increasing model capacity/complexity beyond the point of interpolation actually results in improved performance. While future investigations are needed (eg, where exactly this point of interpolation is), these results offer insights into the potential of Neural Networks.

## Obstacles and Outlook

Although Neural Networks clearly represent the state-of-the-art in image analysis and promise to transform the way we interact with and extract information from imaging data, several factors impede their widespread application, both in scientific and clinical practice. These include hard- and software requirements, data availability, explainability/trustworthiness and ultimately also integration into the clinical workflow.

Creating (and training) Neural Networks requires proficiency with one of the existing Deep Learning frameworks, of which arguably Tensorflow (https://www.tensorflow.org/) and PyTorch (https://pytorch.org/) are the 2 most commonly used. While very powerful, these frameworks can also easily overwhelm beginners. To lower the entry threshold, high-level frameworks on top of Tensorflow and PyTorch have been created: Keras (https://keras.io/) and fast.ai (https://www.fast.ai/), which allow training state-of-the-art Neural Network architectures in a few lines of code. Coupled with algorithmic repositories such as ModelHub.ai (http://modelhub.ai/), this relevantly simplifies the application of Neural Networks.

While not exclusive to Neural Networks, their demand for large amounts of (labeled) training data might deter people from applying them to their research question. Several strategies such as data augmentation or transfer learning exist to ameliorate this dependence on large datasets as discussed in the section above. In addition, well-curated public datasets such as The Cancer Imaging Archive^18^ provide additional data and also increase the data heterogeneity, potentially making the resulting algorithms more robust.

Neural Networks have drawn criticism for their “black box” nature, making it difficult to understand how they arrive at their conclusions. This is considered to be in contrast with traditional machine learning algorithms such as regression models or Random Forest ensembles, which all provide some sort of “importance” measure of the variables used. Nonetheless, a variety of strategies has been proposed to “illuminate the black box,” ranging from visualization of the final feature layer of a CNN,^19^ to visual or textual explanations as exemplified in Grad-CAM^20^ or spine reporting.^21^

Ultimately, especially for a more widespread clinical adoption of these techniques, integration into the routine workflow (and the Picture Archiving and Communication System) is necessary.

To effectively lower many of these barriers, software projects such as the BraTS (Brain Tumor Segmentation) toolkit^22^ facilitate more widespread use of Deep Learning techniques in scientific (and potentially also clinical) practice by offering well integrated, easy-to-use workflows from image preprocessing to segmentation (to analysis eventually).

## Funding

B.W. and B.M. are supported through the Deutsche Forschungsgemeinschaft (DFG), SFB-824, project B12.

**Conflict of interest statement**. None declared.
